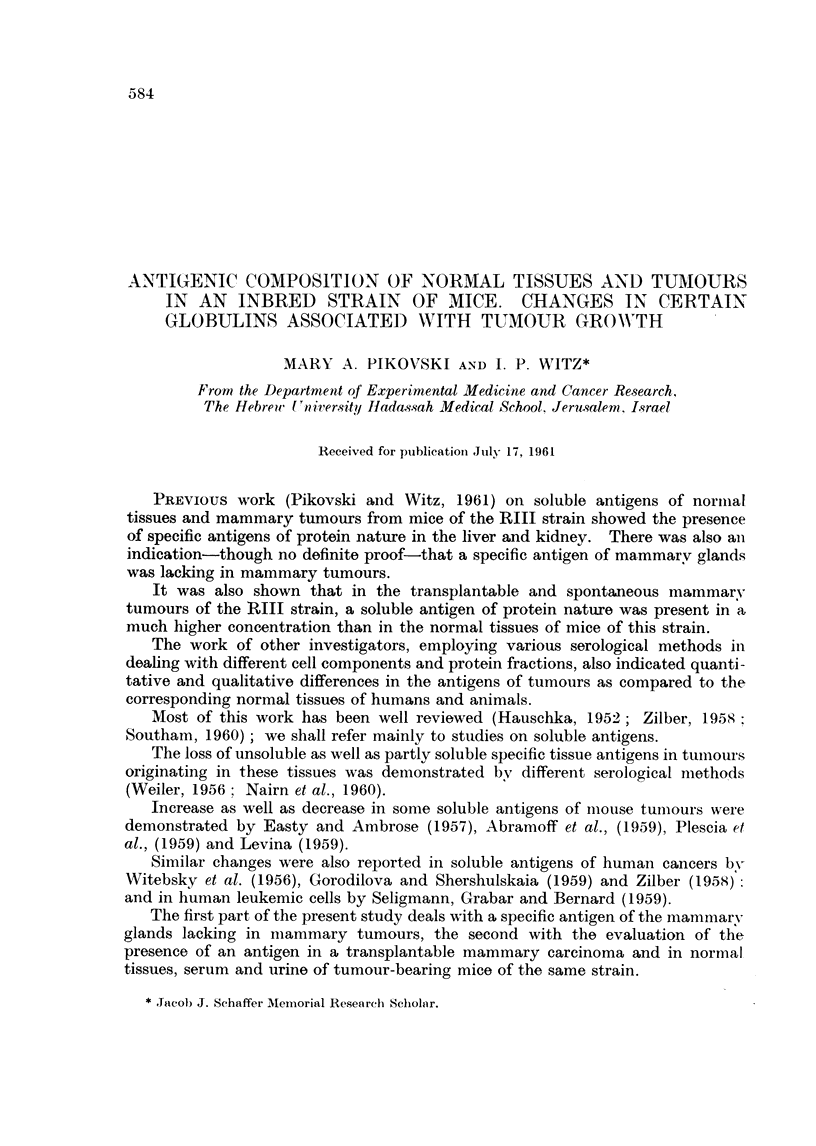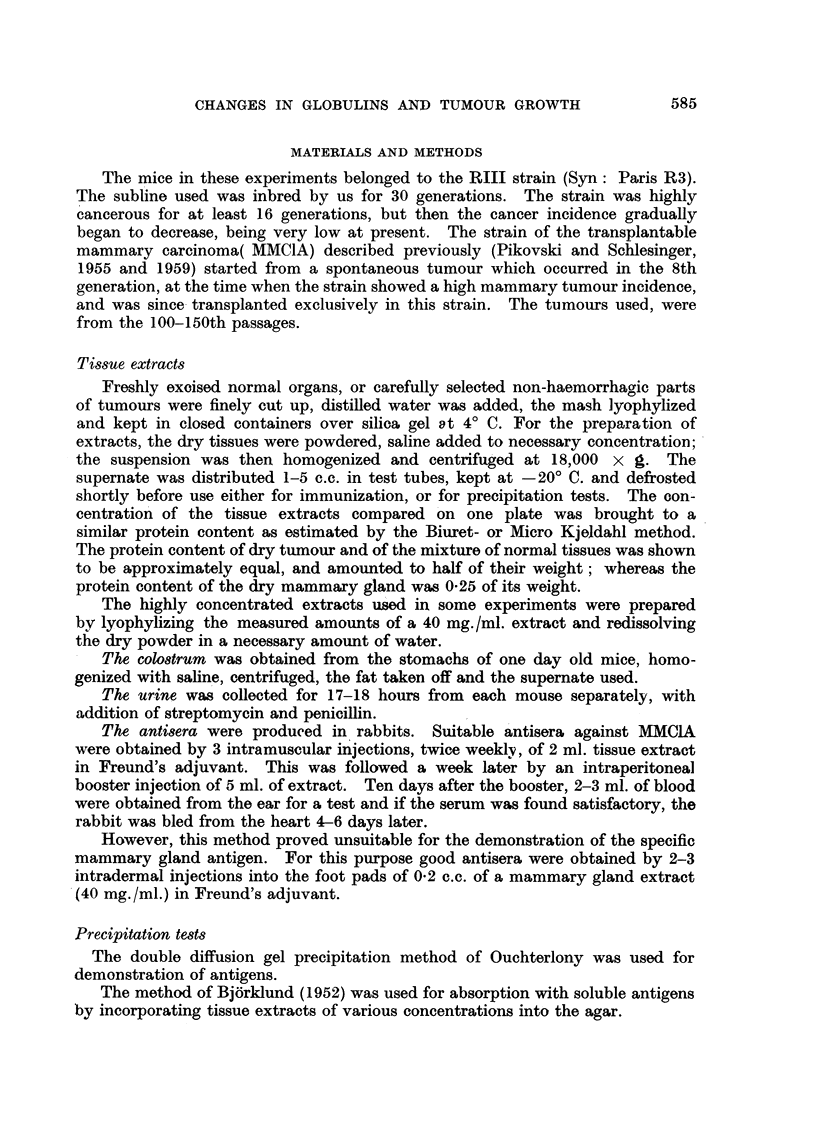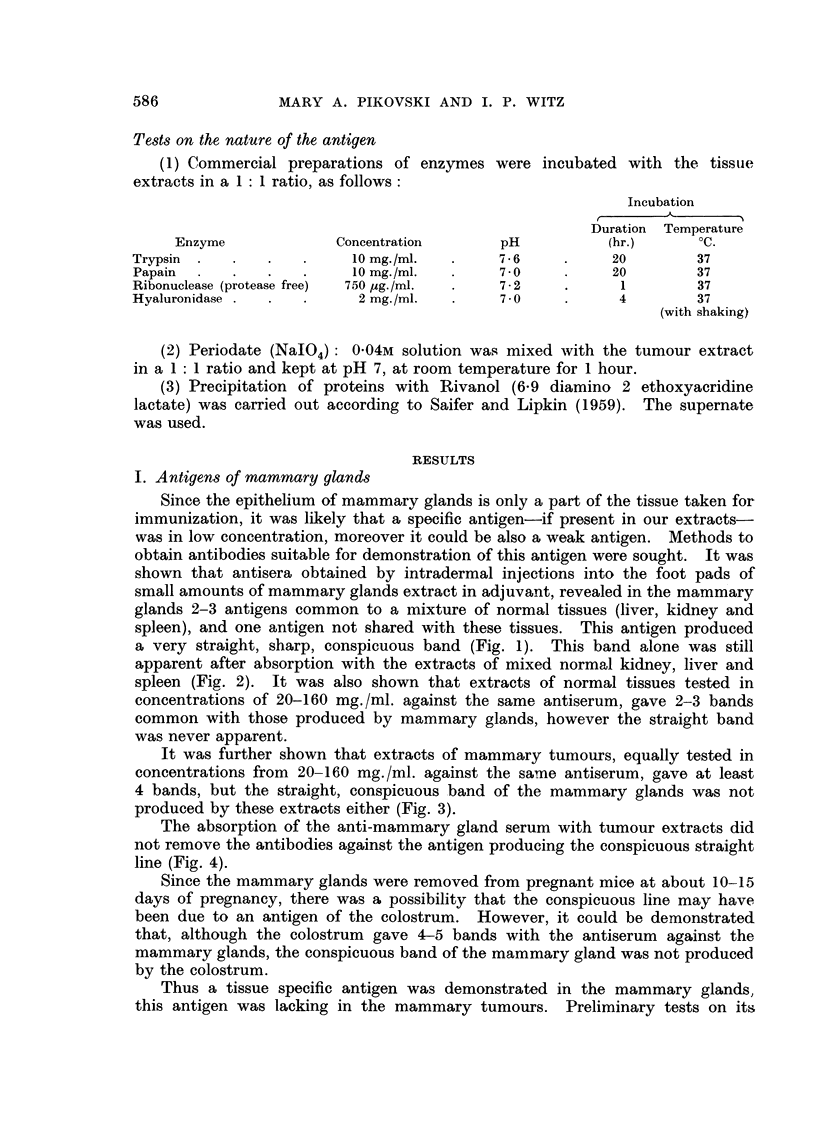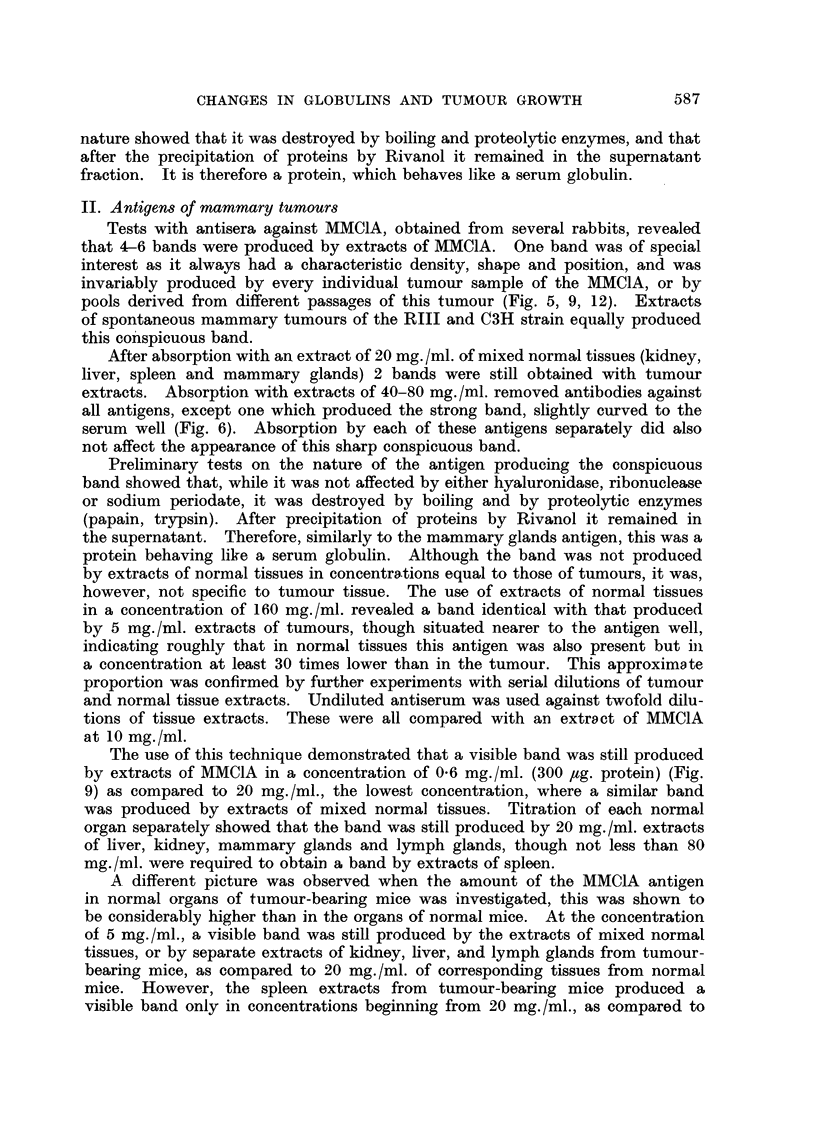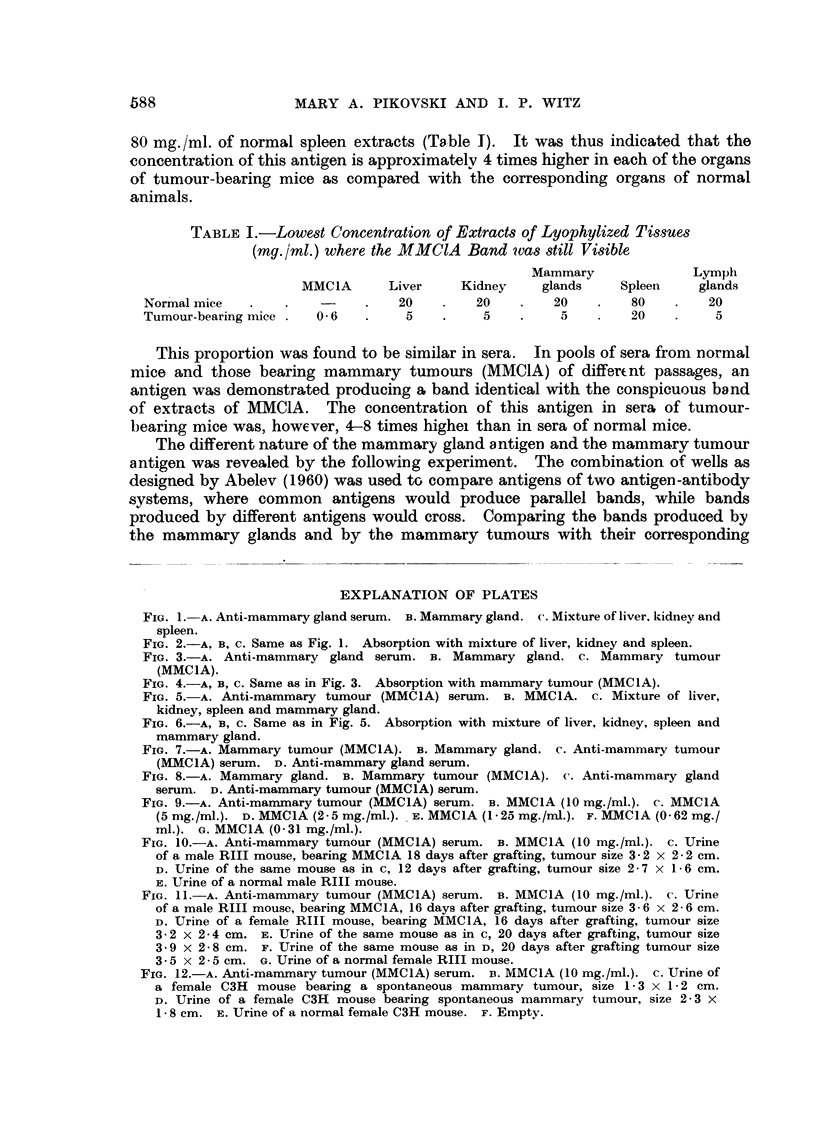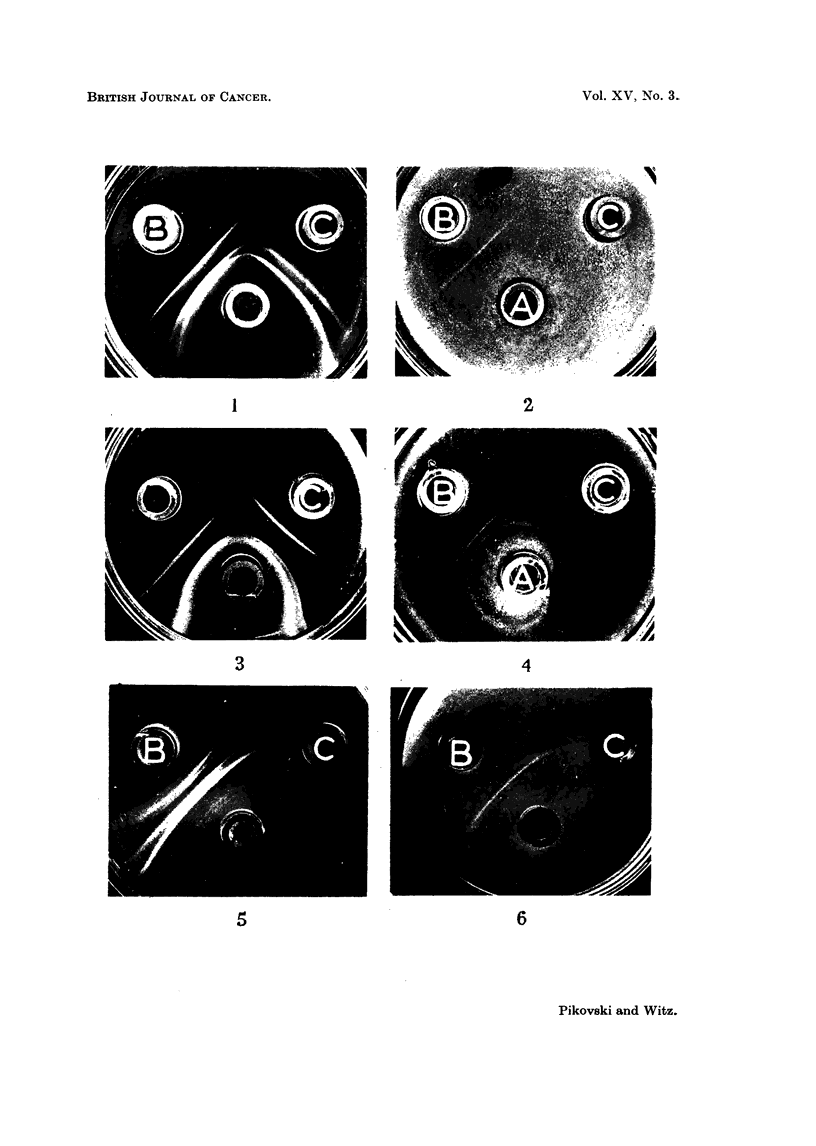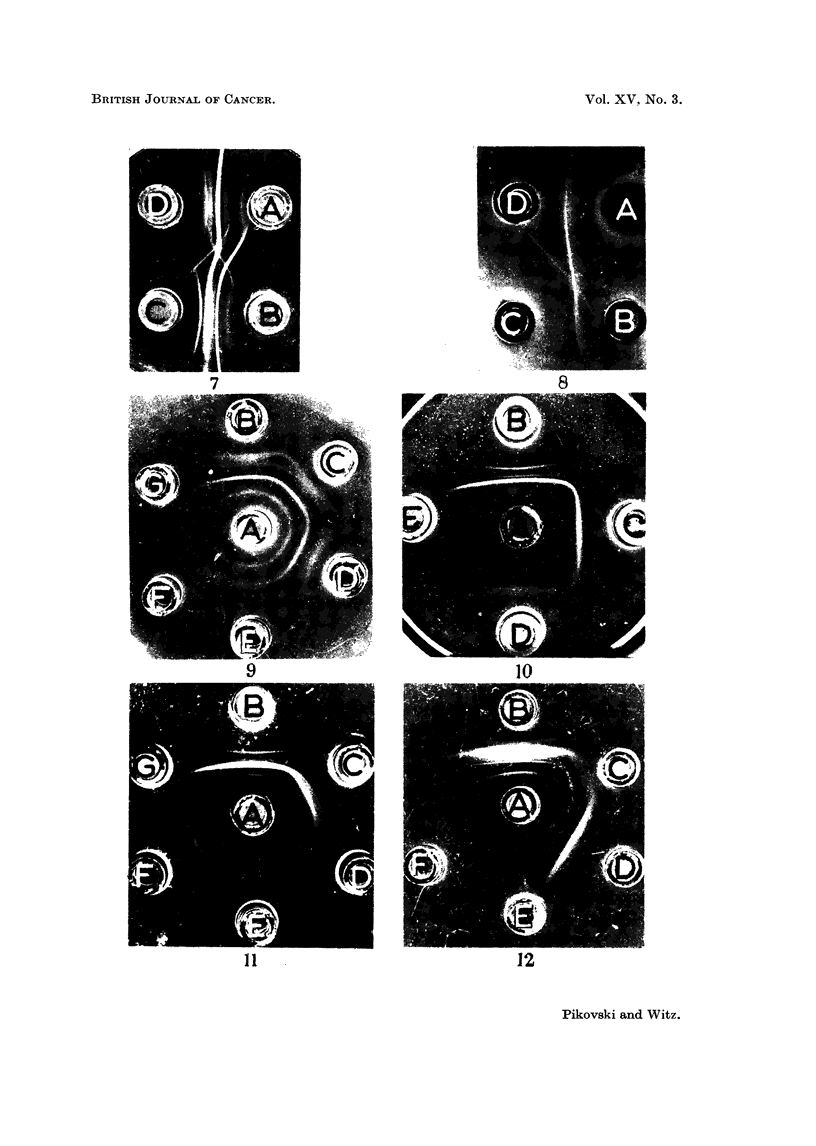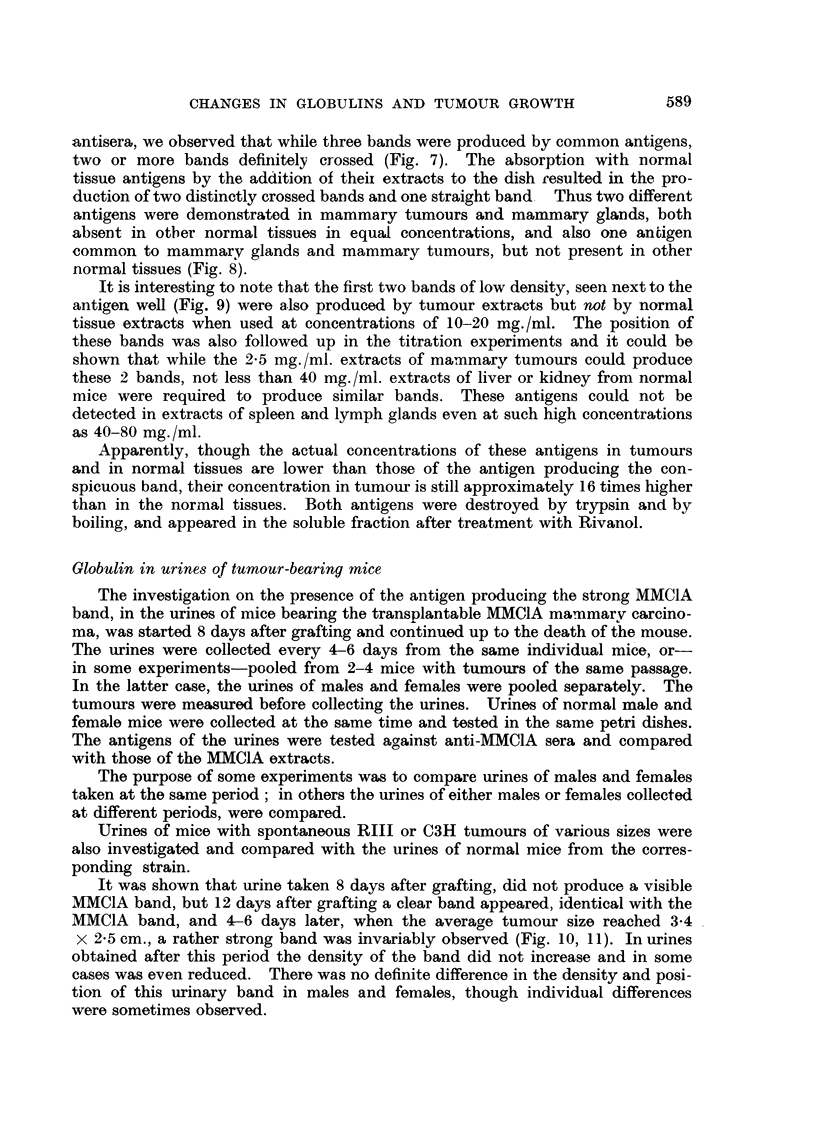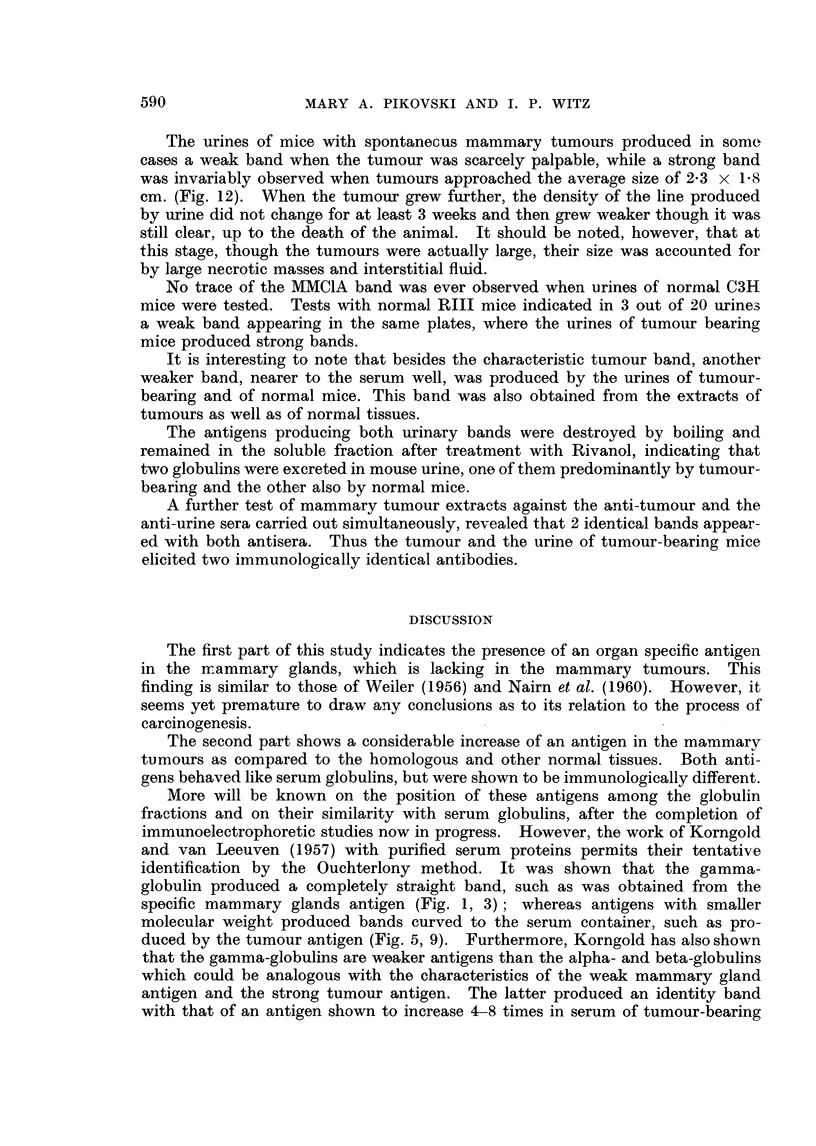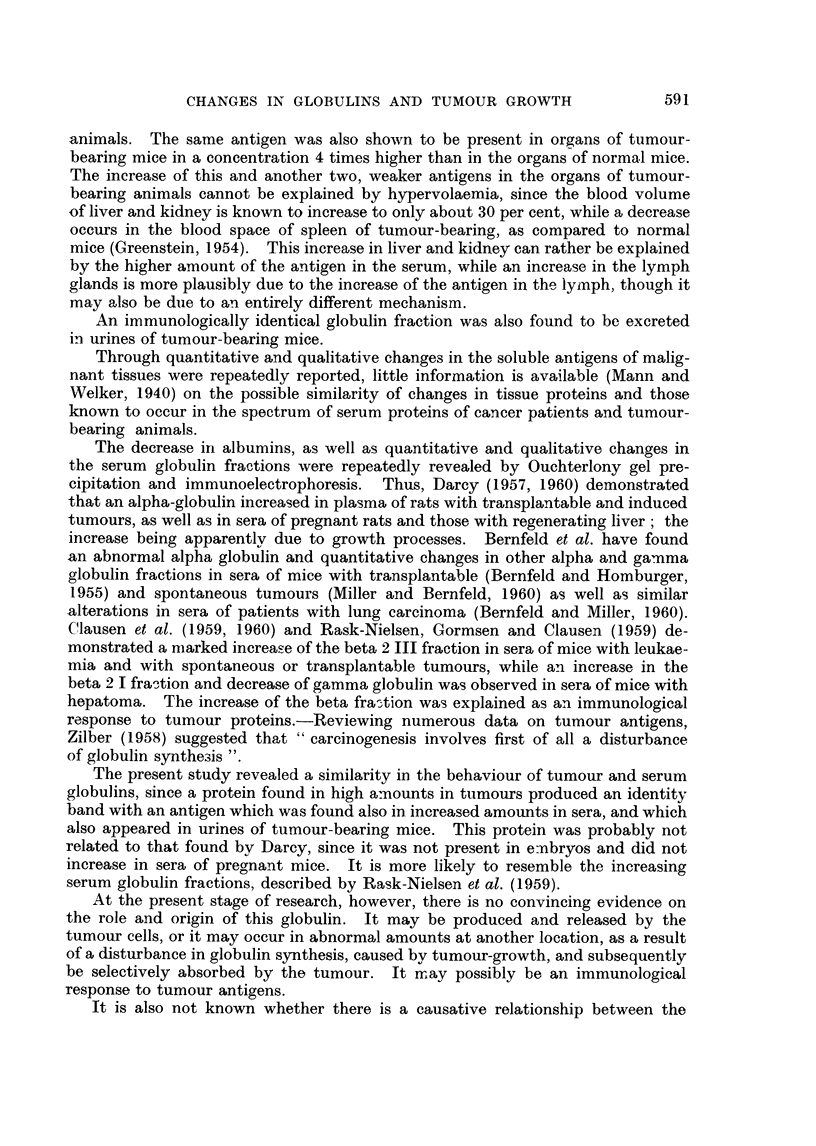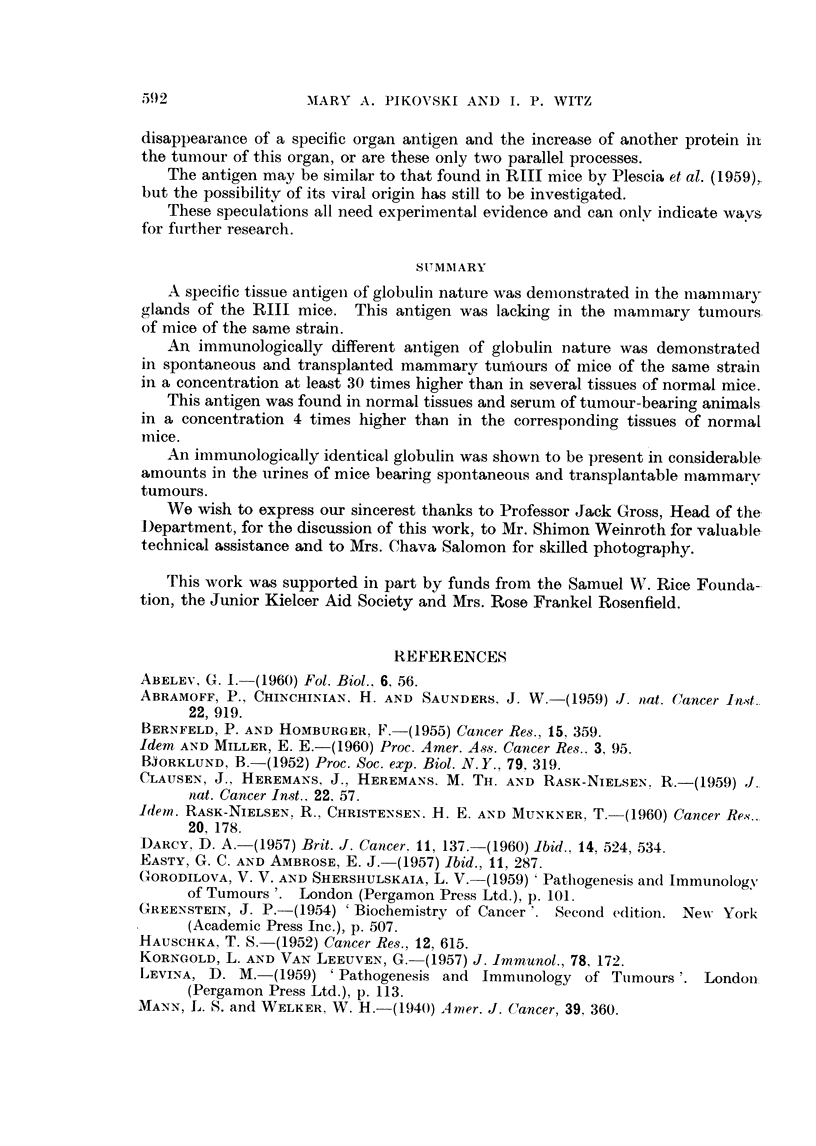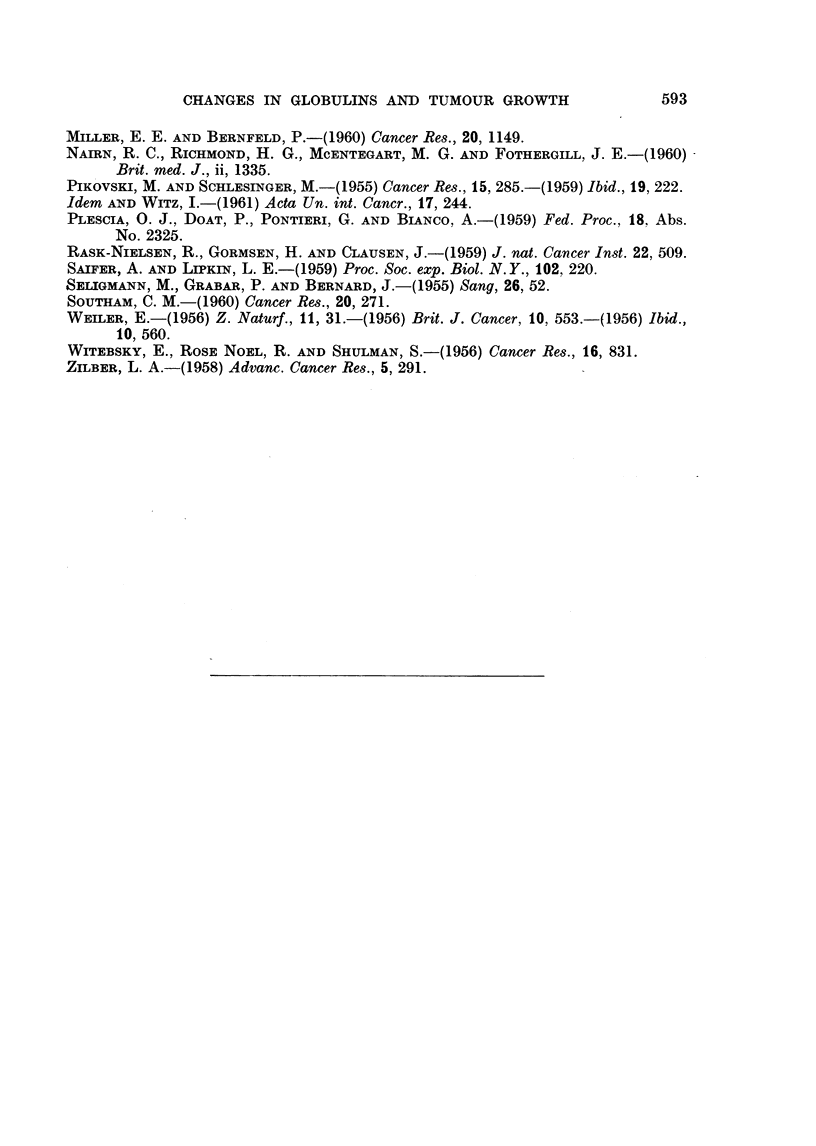# Antigenic Composition of Normal Tissues and Tumours in an Inbred Strain of Mice. Changes in Certain Globulins Associated with Tumour Growth

**DOI:** 10.1038/bjc.1961.68

**Published:** 1961-09

**Authors:** Mary A. Pikovski, I. P. Witz

## Abstract

**Images:**


### ANTIGENIC COMPOSITION OF NORMAL TISSUES AND TUMOURS IN AN INBRED STRAIN OF MICE. CHANGES IN CERTAIN GLOBULINS ASSOCIATED WITH TUMOUR GROWTH

MARY A. PIKOVSKI AND I. P. WITZ*

*From the Department of Experimental Medicine and Cancer Research, The Hebrew University Hadassah Medical School, Jerusalem, Israel*

Received for publication July 17, 1961

PREVIOUS work (Pikovski and Witz, 1961) on soluble antigens of normal tissues and mammary tumours from mice of the RIII strain showed the presence of specific antigens of protein nature in the liver and kidney. There was also an indication—though no definite proof—that a specific antigen of mammary glands was lacking in mammary tumours.

It was also shown that in the transplantable and spontaneous mammary tumours of the RIII strain, a soluble antigen of protein nature was present in a much higher concentration than in the normal tissues of mice of this strain.

The work of other investigators, employing various serological methods in dealing with different cell components and protein fractions, also indicated quantitative and qualitative differences in the antigens of tumours as compared to the corresponding normal tissues of humans and animals.

Most of this work has been well reviewed (Hauschka, 1952 ; Zilber, 1958 ; Southam, 1960) ; we shall refer mainly to studies on soluble antigens.

The loss of unsoluble as well as partly soluble specific tissue antigens in tumours originating in these tissues was demonstrated by different serological methods (Weiler, 1956 ; Nairn *et al.*, 1960).

Increase as well as decrease in some soluble antigens of mouse tumours were demonstrated by Easty and Ambrose (1957), Abramoff *et al.*, (1959), Plescia *et al.*, (1959) and Levina (1959).

Similar changes were also reported in soluble antigens of human cancers by Witebsky *et al.* (1956), Gorodilova and Shershulskaia (1959) and Zilber (1958) ; and in human leukemic cells by Seligmann, Grabar and Bernard (1959).

The first part of the present study deals with a specific antigen of the mammary glands lacking in mammary tumours, the second with the evaluation of the presence of an antigen in a transplantable mammary carcinoma and in normal tissues, serum and urine of tumour-bearing mice of the same strain.

* Jacob J. Schaffer Memorial Research Scholar.

### MATERIALS AND METHODS

The mice in these experiments belonged to the RIII strain (Syn : Paris R3). The subline used was inbred by us for 30 generations. The strain was highly cancerous for at least 16 generations, but then the cancer incidence gradually began to decrease, being very low at present. The strain of the transplantable mammary carcinoma( MMClA) described previously (Pikovski and Schlesinger, 1955 and 1959) started from a spontaneous tumour which occurred in the 8th generation, at the time when the strain showed a high mammary tumour incidence, and was since transplanted exclusively in this strain. The tumours used, were from the 100–150th passages.

### *Tissue extracts*

Freshly excised normal organs, or carefully selected non-haemorrhagic parts of tumours were finely cut up, distilled water was added, the mash lyophylized and kept in closed containers over silica gel at 4° C. For the preparation of extracts, the dry tissues were powdered, saline added to necessary concentration; the suspension was then homogenized and centrifuged at 18,000 × g. The supernate was distributed 1–5 c.c. in test tubes, kept at —20° C. and defrosted shortly before use either for immunization, or for precipitation tests. The concentration of the tissue extracts compared on one plate was brought to a similar protein content as estimated by the Biuret- or Micro Kjeldahl method. The protein content of dry tumour and of the mixture of normal tissues was shown to be approximately equal, and amounted to half of their weight ; whereas the protein content of the dry mammary gland was 0·25 of its weight.

The highly concentrated extracts used in some experiments were prepared by lyophylizing the measured amounts of a 40 mg./ml. extract and redissolving the dry powder in a necessary amount of water.

*The colostrum* was obtained from the stomachs of one day old mice, homogenized with saline, centrifuged, the fat taken off and the supernate used.

*The urine* was collected for 17–18 hours from each mouse separately, with addition of streptomycin and penicillin.

*The antisera* were produced in rabbits. Suitable antisera against MMClA were obtained by 3 intramuscular injections, twice weekly, of 2 ml. tissue extract in Freund's adjuvant. This was followed a week later by an intraperitoneal booster injection of 5 ml. of extract. Ten days after the booster, 2–3 ml. of blood were obtained from the ear for a test and if the serum was found satisfactory, the rabbit was bled from the heart 4–6 days later.

However, this method proved unsuitable for the demonstration of the specific mammary gland antigen. For this purpose good antisera were obtained by 2–3 intradermal injections into the foot pads of 0·2 c.c. of a mammary gland extract (40 mg./ml.) in Freund's adjuvant.

### *Precipitation tests*

The double diffusion gel precipitation method of Ouchterlony was used for demonstration of antigens.

The method of Björklund (1952) was used for absorption with soluble antigens by incorporating tissue extracts of various concentrations into the agar.

*Tests on the nature of the antigen*

(1) Commercial preparations of enzymes were incubated with the tissue extracts in a 1 : 1 ratio, as follows :

| Enzyme | Concentration | pH | Incubation Duration (hr.) | Incubation Temperature °C. |
|---|---|---|---|---|
| Trypsin | 10 mg./ml. | 7·6 | 20 | 37 |
| Papain | 10 mg./ml. | 7·0 | 20 | 37 |
| Ribonuclease (protease free) | 750 μg./ml. | 7·2 | 1 | 37 |
| Hyaluronidase | 2 mg./ml. | 7·0 | 4 | 37 (with shaking) |

(2) Periodate ($NaIO_4$) : 0·04M solution was mixed with the tumour extract in a 1 : 1 ratio and kept at pH 7, at room temperature for 1 hour.

(3) Precipitation of proteins with Rivanol (6·9 diamino 2 ethoxyacridine lactate) was carried out according to Saifer and Lipkin (1959). The supernate was used.

### RESULTS

### I. *Antigens of mammary glands*

Since the epithelium of mammary glands is only a part of the tissue taken for immunization, it was likely that a specific antigen—if present in our extracts—was in low concentration, moreover it could be also a weak antigen. Methods to obtain antibodies suitable for demonstration of this antigen were sought. It was shown that antisera obtained by intradermal injections into the foot pads of small amounts of mammary glands extract in adjuvant, revealed in the mammary glands 2–3 antigens common to a mixture of normal tissues (liver, kidney and spleen), and one antigen not shared with these tissues. This antigen produced a very straight, sharp, conspicuous band (Fig. 1). This band alone was still apparent after absorption with the extracts of mixed normal kidney, liver and spleen (Fig. 2). It was also shown that extracts of normal tissues tested in concentrations of 20–160 mg./ml. against the same antiserum, gave 2–3 bands common with those produced by mammary glands, however the straight band was never apparent.

It was further shown that extracts of mammary tumours, equally tested in concentrations from 20–160 mg./ml. against the same antiserum, gave at least 4 bands, but the straight, conspicuous band of the mammary glands was not produced by these extracts either (Fig. 3).

The absorption of the anti-mammary gland serum with tumour extracts did not remove the antibodies against the antigen producing the conspicuous straight line (Fig. 4).

Since the mammary glands were removed from pregnant mice at about 10–15 days of pregnancy, there was a possibility that the conspicuous line may have been due to an antigen of the colostrum. However, it could be demonstrated that, although the colostrum gave 4–5 bands with the antiserum against the mammary glands, the conspicuous band of the mammary gland was not produced by the colostrum.

Thus a tissue specific antigen was demonstrated in the mammary glands, this antigen was lacking in the mammary tumours. Preliminary tests on its nature showed that it was destroyed by boiling and proteolytic enzymes, and that after the precipitation of proteins by Rivanol it remained in the supernatant fraction. It is therefore a protein, which behaves like a serum globulin.

II. *Antigens of mammary tumours*

Tests with antisera against MMClA, obtained from several rabbits, revealed that 4–6 bands were produced by extracts of MMClA. One band was of special interest as it always had a characteristic density, shape and position, and was invariably produced by every individual tumour sample of the MMClA, or by pools derived from different passages of this tumour (Fig. 5, 9, 12). Extracts of spontaneous mammary tumours of the RIII and C3H strain equally produced this conspicuous band.

After absorption with an extract of 20 mg./ml. of mixed normal tissues (kidney, liver, spleen and mammary glands) 2 bands were still obtained with tumour extracts. Absorption with extracts of 40–80 mg./ml. removed antibodies against all antigens, except one which produced the strong band, slightly curved to the serum well (Fig. 6). Absorption by each of these antigens separately did also not affect the appearance of this sharp conspicuous band.

Preliminary tests on the nature of the antigen producing the conspicuous band showed that, while it was not affected by either hyaluronidase, ribonuclease or sodium periodate, it was destroyed by boiling and by proteolytic enzymes (papain, trypsin). After precipitation of proteins by Rivanol it remained in the supernatant. Therefore, similarly to the mammary glands antigen, this was a protein behaving like a serum globulin. Although the band was not produced by extracts of normal tissues in concentrations equal to those of tumours, it was, however, not specific to tumour tissue. The use of extracts of normal tissues in a concentration of 160 mg./ml. revealed a band identical with that produced by 5 mg./ml. extracts of tumours, though situated nearer to the antigen well, indicating roughly that in normal tissues this antigen was also present but in a concentration at least 30 times lower than in the tumour. This approximate proportion was confirmed by further experiments with serial dilutions of tumour and normal tissue extracts. Undiluted antiserum was used against twofold dilutions of tissue extracts. These were all compared with an extract of MMClA at 10 mg./ml.

The use of this technique demonstrated that a visible band was still produced by extracts of MMClA in a concentration of 0·6 mg./ml. (300 μg. protein) (Fig. 9) as compared to 20 mg./ml., the lowest concentration, where a similar band was produced by extracts of mixed normal tissues. Titration of each normal organ separately showed that the band was still produced by 20 mg./ml. extracts of liver, kidney, mammary glands and lymph glands, though not less than 80 mg./ml. were required to obtain a band by extracts of spleen.

A different picture was observed when the amount of the MMClA antigen in normal organs of tumour-bearing mice was investigated, this was shown to be considerably higher than in the organs of normal mice. At the concentration of 5 mg./ml., a visible band was still produced by the extracts of mixed normal tissues, or by separate extracts of kidney, liver, and lymph glands from tumour-bearing mice, as compared to 20 mg./ml. of corresponding tissues from normal mice. However, the spleen extracts from tumour-bearing mice produced a visible band only in concentrations beginning from 20 mg./ml., as compared to

80 mg./ml. of normal spleen extracts (Table I). It was thus indicated that the concentration of this antigen is approximately 4 times higher in each of the organs of tumour-bearing mice as compared with the corresponding organs of normal animals.

TABLE I.—*Lowest Concentration of Extracts of Lyophylized Tissues (mg./ml.) where the MMClA Band was still Visible*

| | MMC1A | Liver | Kidney | Mammary glands | Spleen | Lymph glands |
|---|---|---|---|---|---|---|
| Normal mice | — | 20 | 20 | 20 | 80 | 20 |
| Tumour-bearing mice | 0·6 | 5 | 5 | 5 | 20 | 5 |

This proportion was found to be similar in sera. In pools of sera from normal mice and those bearing mammary tumours (MMClA) of different passages, an antigen was demonstrated producing a band identical with the conspicuous band of extracts of MMClA. The concentration of this antigen in sera of tumour-bearing mice was, however, 4–8 times higher than in sera of normal mice.

The different nature of the mammary gland antigen and the mammary tumour antigen was revealed by the following experiment. The combination of wells as designed by Abelev (1960) was used to compare antigens of two antigen-antibody systems, where common antigens would produce parallel bands, while bands produced by different antigens would cross. Comparing the bands produced by the mammary glands and by the mammary tumours with their corresponding

EXPLANATION OF PLATES

FIG. 1.—A. Anti-mammary gland serum. B. Mammary gland. C. Mixture of liver, kidney and spleen.
FIG. 2.—A, B, C. Same as Fig. 1. Absorption with mixture of liver, kidney and spleen.
FIG. 3.—A. Anti-mammary gland serum. B. Mammary gland. C. Mammary tumour (MMC1A).
FIG. 4.—A, B, C. Same as in Fig. 3. Absorption with mammary tumour (MMC1A).
FIG. 5.—A. Anti-mammary tumour (MMC1A) serum. B. MMC1A. C. Mixture of liver, kidney, spleen and mammary gland.
FIG. 6.—A, B, C. Same as in Fig. 5. Absorption with mixture of liver, kidney, spleen and mammary gland.
FIG. 7.—A. Mammary tumour (MMC1A). B. Mammary gland. C. Anti-mammary tumour (MMC1A) serum. D. Anti-mammary gland serum.
FIG. 8.—A. Mammary gland. B. Mammary tumour (MMC1A). C. Anti-mammary gland serum. D. Anti-mammary tumour (MMC1A) serum.
FIG. 9.—A. Anti-mammary tumour (MMC1A) serum. B. MMC1A (10 mg./ml.). C. MMC1A (5 mg./ml.). D. MMC1A (2·5 mg./ml.). E. MMC1A (1·25 mg./ml.). F. MMC1A (0·62 mg./ml.). G. MMC1A (0·31 mg./ml.).
FIG. 10.—A. Anti-mammary tumour (MMC1A) serum. B. MMC1A (10 mg./ml.). C. Urine of a male RIII mouse, bearing MMC1A 18 days after grafting, tumour size 3·2 × 2·2 cm. D. Urine of the same mouse as in C, 12 days after grafting, tumour size 2·7 × 1·6 cm. E. Urine of a normal male RIII mouse.
FIG. 11.—A. Anti-mammary tumour (MMC1A) serum. B. MMC1A (10 mg./ml.). C. Urine of a male RIII mouse, bearing MMC1A, 16 days after grafting, tumour size 3·6 × 2·6 cm. D. Urine of a female RIII mouse, bearing MMC1A, 16 days after grafting, tumour size 3·2 × 2·4 cm. E. Urine of the same mouse as in C, 20 days after grafting, tumour size 3·9 × 2·8 cm. F. Urine of the same mouse as in D, 20 days after grafting tumour size 3·5 × 2·5 cm. G. Urine of a normal female RIII mouse.
FIG. 12.—A. Anti-mammary tumour (MMC1A) serum. B. MMC1A (10 mg./ml.). C. Urine of a female C3H mouse bearing a spontaneous mammary tumour, size 1·3 × 1·2 cm. D. Urine of a female C3H mouse bearing spontaneous mammary tumour, size 2·3 × 1·8 cm. E. Urine of a normal female C3H mouse. F. Empty.

1

2

3

4

5

6

7

8

9

10

11

12

antisera, we observed that while three bands were produced by common antigens, two or more bands definitely crossed (Fig. 7). The absorption with normal tissue antigens by the addition of their extracts to the dish resulted in the production of two distinctly crossed bands and one straight band Thus two different antigens were demonstrated in mammary tumours and mammary glands, both absent in other normal tissues in equal concentrations, and also one antigen common to mammary glands and mammary tumours, but not present in other normal tissues (Fig. 8).

It is interesting to note that the first two bands of low density, seen next to the antigen well (Fig. 9) were also produced by tumour extracts but *not* by normal tissue extracts when used at concentrations of 10–20 mg./ml. The position of these bands was also followed up in the titration experiments and it could be shown that while the 2·5 mg./ml. extracts of mammary tumours could produce these 2 bands, not less than 40 mg./ml. extracts of liver or kidney from normal mice were required to produce similar bands. These antigens could not be detected in extracts of spleen and lymph glands even at such high concentrations as 40–80 mg./ml.

Apparently, though the actual concentrations of these antigens in tumours and in normal tissues are lower than those of the antigen producing the conspicuous band, their concentration in tumour is still approximately 16 times higher than in the normal tissues. Both antigens were destroyed by trypsin and by boiling, and appeared in the soluble fraction after treatment with Rivanol.

*Globulin in urines of tumour-bearing mice*

The investigation on the presence of the antigen producing the strong MMClA band, in the urines of mice bearing the transplantable MMClA mammary carcinoma, was started 8 days after grafting and continued up to the death of the mouse. The urines were collected every 4–6 days from the same individual mice, or—in some experiments—pooled from 2–4 mice with tumours of the same passage. In the latter case, the urines of males and females were pooled separately. The tumours were measured before collecting the urines. Urines of normal male and female mice were collected at the same time and tested in the same petri dishes. The antigens of the urines were tested against anti-MMClA sera and compared with those of the MMClA extracts.

The purpose of some experiments was to compare urines of males and females taken at the same period ; in others the urines of either males or females collected at different periods, were compared.

Urines of mice with spontaneous RIII or C3H tumours of various sizes were also investigated and compared with the urines of normal mice from the corresponding strain.

It was shown that urine taken 8 days after grafting, did not produce a visible MMClA band, but 12 days after grafting a clear band appeared, identical with the MMClA band, and 4–6 days later, when the average tumour size reached 3·4 × 2·5 cm., a rather strong band was invariably observed (Fig. 10, 11). In urines obtained after this period the density of the band did not increase and in some cases was even reduced. There was no definite difference in the density and position of this urinary band in males and females, though individual differences were sometimes observed.

The urines of mice with spontaneous mammary tumours produced in some cases a weak band when the tumour was scarcely palpable, while a strong band was invariably observed when tumours approached the average size of 2·3 × 1·8 cm. (Fig. 12). When the tumour grew further, the density of the line produced by urine did not change for at least 3 weeks and then grew weaker though it was still clear, up to the death of the animal. It should be noted, however, that at this stage, though the tumours were actually large, their size was accounted for by large necrotic masses and interstitial fluid.

No trace of the MMC1A band was ever observed when urines of normal C3H mice were tested. Tests with normal RIII mice indicated in 3 out of 20 urines a weak band appearing in the same plates, where the urines of tumour bearing mice produced strong bands.

It is interesting to note that besides the characteristic tumour band, another weaker band, nearer to the serum well, was produced by the urines of tumour-bearing and of normal mice. This band was also obtained from the extracts of tumours as well as of normal tissues.

The antigens producing both urinary bands were destroyed by boiling and remained in the soluble fraction after treatment with Rivanol, indicating that two globulins were excreted in mouse urine, one of them predominantly by tumour-bearing and the other also by normal mice.

A further test of mammary tumour extracts against the anti-tumour and the anti-urine sera carried out simultaneously, revealed that 2 identical bands appeared with both antisera. Thus the tumour and the urine of tumour-bearing mice elicited two immunologically identical antibodies.

### DISCUSSION

The first part of this study indicates the presence of an organ specific antigen in the mammary glands, which is lacking in the mammary tumours. This finding is similar to those of Weiler (1956) and Nairn *et al.* (1960). However, it seems yet premature to draw any conclusions as to its relation to the process of carcinogenesis.

The second part shows a considerable increase of an antigen in the mammary tumours as compared to the homologous and other normal tissues. Both antigens behaved like serum globulins, but were shown to be immunologically different.

More will be known on the position of these antigens among the globulin fractions and on their similarity with serum globulins, after the completion of immunoelectrophoretic studies now in progress. However, the work of Korngold and van Leeuven (1957) with purified serum proteins permits their tentative identification by the Ouchterlony method. It was shown that the gamma-globulin produced a completely straight band, such as was obtained from the specific mammary glands antigen (Fig. 1, 3); whereas antigens with smaller molecular weight produced bands curved to the serum container, such as produced by the tumour antigen (Fig. 5, 9). Furthermore, Korngold has also shown that the gamma-globulins are weaker antigens than the alpha- and beta-globulins which could be analogous with the characteristics of the weak mammary gland antigen and the strong tumour antigen. The latter produced an identity band with that of an antigen shown to increase 4–8 times in serum of tumour-bearing animals. The same antigen was also shown to be present in organs of tumour-bearing mice in a concentration 4 times higher than in the organs of normal mice. The increase of this and another two, weaker antigens in the organs of tumour-bearing animals cannot be explained by hypervolaemia, since the blood volume of liver and kidney is known to increase to only about 30 per cent, while a decrease occurs in the blood space of spleen of tumour-bearing, as compared to normal mice (Greenstein, 1954). This increase in liver and kidney can rather be explained by the higher amount of the antigen in the serum, while an increase in the lymph glands is more plausibly due to the increase of the antigen in the lymph, though it may also be due to an entirely different mechanism.

An immunologically identical globulin fraction was also found to be excreted in urines of tumour-bearing mice.

Through quantitative and qualitative changes in the soluble antigens of malignant tissues were repeatedly reported, little information is available (Mann and Welker, 1940) on the possible similarity of changes in tissue proteins and those known to occur in the spectrum of serum proteins of cancer patients and tumour-bearing animals.

The decrease in albumins, as well as quantitative and qualitative changes in the serum globulin fractions were repeatedly revealed by Ouchterlony gel precipitation and immunoelectrophoresis. Thus, Darcy (1957, 1960) demonstrated that an alpha-globulin increased in plasma of rats with transplantable and induced tumours, as well as in sera of pregnant rats and those with regenerating liver ; the increase being apparently due to growth processes. Bernfeld *et al.* have found an abnormal alpha globulin and quantitative changes in other alpha and gamma globulin fractions in sera of mice with transplantable (Bernfeld and Homburger, 1955) and spontaneous tumours (Miller and Bernfeld, 1960) as well as similar alterations in sera of patients with lung carcinoma (Bernfeld and Miller, 1960). Clausen *et al.* (1959, 1960) and Rask-Nielsen, Gormsen and Clausen (1959) demonstrated a marked increase of the beta 2 III fraction in sera of mice with leukaemia and with spontaneous or transplantable tumours, while an increase in the beta 2 I fraction and decrease of gamma globulin was observed in sera of mice with hepatoma. The increase of the beta fraction was explained as an immunological response to tumour proteins.—Reviewing numerous data on tumour antigens, Zilber (1958) suggested that " carcinogenesis involves first of all a disturbance of globulin synthesis ".

The present study revealed a similarity in the behaviour of tumour and serum globulins, since a protein found in high amounts in tumours produced an identity band with an antigen which was found also in increased amounts in sera, and which also appeared in urines of tumour-bearing mice. This protein was probably not related to that found by Darcy, since it was not present in embryos and did not increase in sera of pregnant mice. It is more likely to resemble the increasing serum globulin fractions, described by Rask-Nielsen *et al.* (1959).

At the present stage of research, however, there is no convincing evidence on the role and origin of this globulin. It may be produced and released by the tumour cells, or it may occur in abnormal amounts at another location, as a result of a disturbance in globulin synthesis, caused by tumour-growth, and subsequently be selectively absorbed by the tumour. It may possibly be an immunological response to tumour antigens.

It is also not known whether there is a causative relationship between the disappearance of a specific organ antigen and the increase of another protein in the tumour of this organ, or are these only two parallel processes.

The antigen may be similar to that found in RIII mice by Plescia *et al.* (1959), but the possibility of its viral origin has still to be investigated.

These speculations all need experimental evidence and can only indicate ways for further research.

### SUMMARY

A specific tissue antigen of globulin nature was demonstrated in the mammary glands of the RIII mice. This antigen was lacking in the mammary tumours of mice of the same strain.

An immunologically different antigen of globulin nature was demonstrated in spontaneous and transplanted mammary tumours of mice of the same strain in a concentration at least 30 times higher than in several tissues of normal mice.

This antigen was found in normal tissues and serum of tumour-bearing animals in a concentration 4 times higher than in the corresponding tissues of normal mice.

An immunologically identical globulin was shown to be present in considerable amounts in the urines of mice bearing spontaneous and transplantable mammary tumours.

We wish to express our sincerest thanks to Professor Jack Gross, Head of the Department, for the discussion of this work, to Mr. Shimon Weinroth for valuable technical assistance and to Mrs. Chava Salomon for skilled photography.

This work was supported in part by funds from the Samuel W. Rice Foundation, the Junior Kielcer Aid Society and Mrs. Rose Frankel Rosenfield.

### REFERENCES

ABELEV, G. I.—(1960) *Fol. Biol.*, **6**, 56.

ABRAMOFF, P., CHINCHINIAN, H. AND SAUNDERS, J. W.—(1959) *J. nat. Cancer Inst.*, **22**, 919.

BERNFELD, P. AND HOMBURGER, F.—(1955) *Cancer Res.*, **15**, 359.

*Idem* AND MILLER, E. E.—(1960) *Proc. Amer. Ass. Cancer Res.*, **3**, 95.

BJÖRKLUND, B.—(1952) *Proc. Soc. exp. Biol. N.Y.*, **79**, 319.

CLAUSEN, J., HEREMANS, J., HEREMANS, M. TH. AND RASK-NIELSEN, R.—(1959) *J. nat. Cancer Inst.*, **22**, 57.

*Idem*, RASK-NIELSEN, R., CHRISTENSEN, H. E. AND MUNKNER, T.—(1960) *Cancer Res.*, **20**, 178.

DARCY, D. A.—(1957) *Brit. J. Cancer*, **11**, 137.—(1960) *Ibid.*, **14**, 524, 534.

EASTY, G. C. AND AMBROSE, E. J.—(1957) *Ibid.*, **11**, 287.

GORODILOVA, V. V. AND SHERSHULSKAIA, L. V.—(1959) ' Pathogenesis and Immunology of Tumours '. London (Pergamon Press Ltd.), p. 101.

GREENSTEIN, J. P.—(1954) ' Biochemistry of Cancer '. Second edition. New York (Academic Press Inc.), p. 507.

HAUSCHKA, T. S.—(1952) *Cancer Res.*, **12**, 615.

KORNGOLD, L. AND VAN LEEUVEN, G.—(1957) *J. Immunol.*, **78**, 172.

LEVINA, D. M.—(1959) ' Pathogenesis and Immunology of Tumours '. London (Pergamon Press Ltd.), p. 113.

MANN, L. S. and WELKER, W. H.—(1940) *Amer. J. Cancer*, **39**, 360.

MILLER, E. E. AND BERNFELD, P.—(1960) *Cancer Res.*, **20**, 1149.
NAIRN, R. C., RICHMOND, H. G., MCENTEGART, M. G. AND FOTHERGILL, J. E.—(1960) *Brit. med. J.*, ii, 1335.
PIKOVSKI, M. AND SCHLESINGER, M.—(1955) *Cancer Res.*, **15**, 285.—(1959) *Ibid.*, **19**, 222.
*Idem* AND WITZ, I.—(1961) *Acta Un. int. Cancr.*, **17**, 244.
PLESCIA, O. J., DOAT, P., PONTIERI, G. AND BIANCO, A.—(1959) *Fed. Proc.*, **18**, Abs. No. 2325.
RASK-NIELSEN, R., GORMSEN, H. AND CLAUSEN, J.—(1959) *J. nat. Cancer Inst.* **22**, 509.
SAIFER, A. AND LIPKIN, L. E.—(1959) *Proc. Soc. exp. Biol. N.Y.*, **102**, 220.
SELIGMANN, M., GRABAR, P. AND BERNARD, J.—(1955) *Sang*, **26**, 52.
SOUTHAM, C. M.—(1960) *Cancer Res.*, **20**, 271.
WEILER, E.—(1956) *Z. Naturf.*, **11**, 31.—(1956) *Brit. J. Cancer*, **10**, 553.—(1956) *Ibid.*, **10**, 560.
WITEBSKY, E., ROSE NOEL, R. AND SHULMAN, S.—(1956) *Cancer Res.*, **16**, 831.
ZILBER, L. A.—(1958) *Advanc. Cancer Res.*, **5**, 291.